# Nephrotic Syndrome: Genetics, Mechanism, and Therapies

**DOI:** 10.1155/2018/6215946

**Published:** 2018-01-28

**Authors:** Jae Il Shin, Andreas Kronbichler, Jun Oh, Björn Meijers

**Affiliations:** ^1^Institute of Kidney Disease Research, Department of Pediatrics, Yonsei University College of Medicine and Department of Pediatric Nephrology, Severance Children's Hospital, Seoul, Republic of Korea; ^2^Department of Internal Medicine IV (Nephrology and Hypertension), Medical University Innsbruck, Innsbruck, Austria; ^3^Pediatric Nephrology, University Medical Center Hamburg-Eppendorf, Hamburg, Germany; ^4^Department of Microbiology and Immunology, KU Leuven and Department of Nephrology, UZ Leuven, Leuven, Belgium

Nephrotic syndrome (NS) is characterized by massive proteinuria, hypoalbuminemia, and hypercholesterolemia. Minimal change nephrotic syndrome (MCNS) is the most common form of NS in children and membranous nephropathy (MN) is more common in adults. Focal segmental glomerulosclerosis (FSGS) is one of the primary glomerular disorders in both children and adults which frequently shows steroid resistance and can progress to end-stage renal failure. Recently, there have been advances in the fields of pathogenesis and treatment of various kinds of NS. In this special issue, several investigators reviewed the recent advances or presented the experimental works regarding the pathogenesis and treatment of NS.

The etiopathogenesis of NS is incompletely understood. To understand the pathogenesis of primary MN, A. Kronbichler et al. reviewed the discovery of several antibodies and genetics which contributed to an increased understanding of MN, such as antibodies against the M-type phospholipase A2 receptor (PLA2R), several risk alleles related to the PLA2R1 gene, and thrombospondin type 1 domain-containing 7A (THSD7A) antibodies.

To understand the genetic causes of NS, M. S. Guaragna et al. reviewed the list of NPHS2 mutations reported between June 2013 and February 2017, with a closer look to mutations occurring in Latin American countries, emphasizing the importance of implementing the molecular evaluation of NS. To understand the pathogenesis of NS, P. Zapata-Benavides et al. measured different expression levels and protein localization of WT1 in patients with steroid-sensitive and steroid-resistant NS and investigated its relationship with miR-15a, miR-16-1, and miR-193a, which modulate the WT1 expression in other models.

V. J. Savin et al. provide an overview of the circulating permeability factors in primary FSGS that have been implicated in the pathogenesis of the potential recurrence in renal allografts after kidney transplantation, focusing on cardiotrophin-like cytokine factor-1 (CLCF-1); they found and suggested therapies specific to CLCF-1, including potential use of cytokine receptor-like factor (CRLF-1) and inhibition of Janus kinase 2.

Regarding the treatment of NS, S. Thalgahagoda et al. presented their experiences on administering the anticancer drug, vincristine, in patients with steroid-resistant NS and suggested that vincristine could be a potential alternative treatment of steroid-resistant NS patients. Rituximab, a chimeric B-cell depleting anti-CD20 antibody, has emerged in the last decade as a treatment option for patients with primary glomerular diseases and M. Rudnicki introduced the data on the use of rituximab in membranoproliferative glomerulonephritis (MPGN), C3 glomerulonephritis (C3GN), and dense deposit disease (DDD) by reviewing the published case reports and retrospective case series.

To provide insight into potential new targets for the treatment of NS from recent basic science publications, E. Königshausen and L. Sellin reviewed recent advances in the treatment of primary causes of NS (idiopathic MN, MCNS, and FSGS) since the publication of the KDIGO guidelines in 2012 and introduced the ongoing RCTs for these diseases that will provide further information on the effectiveness of different treatment options for the causative disease.

Articles published in this special issue covered the various fields of NS such as pathophysiologic mechanisms and treatment options. Understanding theses various faces of NS will lead to the better patient treatment and outcome.

*Jae Il Shin* *Andreas Kronbichler* *Jun Oh* *Björn Meijers*

